# Recurrent giant neurofibroma of the posterior neck and upper back with failed primary grafting and salvage reconstruction: a case report

**DOI:** 10.3389/fsurg.2026.1876161

**Published:** 2026-07-24

**Authors:** Bo Zhou, Bo Liu, Tianyao Lan

**Affiliations:** 1 Yichang Central People's Hospital, Yichang, China; 2 The First College of Clinical Medical Science, China Three Gorges University, Yichang, China; 3 Department of Burn and Plastic Surgery, Beihai People's Hospital, the Ninth Affiliated Hospital of Guangxi Medical University, Beihai, Guangxi Zhuang Autonomous Region, China

**Keywords:** case report, hemangioma, negative-pressure wound therapy, neurofibroma, neurofibromatosis type 1, split-thickness skin graft, wound reconstruction

## Abstract

**Background:**

Giant or recurrent neurofibromas associated with neurofibromatosis type 1 (NF1) may mimic vascular tumors and create large reconstructive defects, particularly in mobile regions. We report a recurrent posterior neck and upper-back neurofibroma with failure of primary grafting and subsequent salvage reconstruction.

**Case presentation:**

A 23-year-old woman had previously undergone an aborted operation for a lesion diagnosed as hemangioma; postoperative pathology showed neurofibroma. The recurrent mass was excised along the superficial aspect of the deep fascia with an estimated blood loss of approximately 100 mL, leaving a defect involving about 7% of total body surface area. A split-thickness graft harvested from clinically preserved skin overlying the tumor failed after subgraft seroma formation and Klebsiella pneumoniae infection, with approximately 20% graft take. Salvage treatment consisted of repeat debridement, culture-directed infection control, a 1:3 meshed split-thickness graft from the thigh, negative-pressure wound therapy adapted into separate neck and back dressing zones, and postoperative immobilization. Graft take was complete. At 6 months, no recurrence was detected, neck motion was preserved, the Vancouver Scar Scale score was 3, and the Neck Disability Index score was 5.

**Conclusions:**

This case emphasizes accurate diagnosis, careful selection of graft donor skin, adequate wound-bed preparation, infection control, secure graft fixation, and immobilization in a high-mobility region. Dividing the negative-pressure dressing into separate zones was a practical modification for this wound rather than a novel reconstructive principle, and broader recommendations cannot be made from a single case.

## Introduction

Neurofibromatosis type 1 is an autosomal dominant tumor-predisposition disorder with cutaneous, neurologic, skeletal, and soft-tissue manifestations (1–8). Plexiform or diffuse neurofibromas may cause disfigurement, pain, functional impairment, and concern for malignant transformation. MEK-inhibitor therapy has expanded treatment options for selected inoperable plexiform neurofibromas, but surgery remains important for resectable, symptomatic, recurrent, or deforming lesions (4, 6, 9–14).

Large NF1-associated tumors can be mistaken for vascular lesions when imaging shows prominent or disorganized vessels (15). Surgery may be complicated by vascular fragility, prior scarring, recurrence, and the need to reconstruct a broad irregular defect (11, 12, 16, 17). Skin overlying a tumor has occasionally been reused to reduce donor-site morbidity, but its biological and oncologic suitability cannot be assumed when the lesion is infiltrative or involves the dermis and subcutaneous tissue (16, 18).

Here, we describe a young woman with a recurrent giant neurofibroma of the posterior neck and upper back that was initially misdiagnosed as hemangioma. We focus on three clinically relevant issues: diagnostic pitfalls, blood-sparing resection along an appropriate anatomic plane, and salvage reconstruction after failure of tumor-surface recycled grafting.

## Case presentation

### History and clinical findings

A 23-year-old woman was admitted in September 2025 with an 11-year history of a posterior neck mass and a 2-year history of recurrent enlargement extending to the upper back. Approximately 8 years earlier, surgery had been attempted elsewhere under a diagnosis of hemangioma but was stopped because of marked bleeding. Postoperative histopathology reportedly showed neurofibroma, and no standardized follow-up was undertaken. The lesion enlarged again from 2023 and caused local traction without ulceration, severe pain, or limb dysfunction.

Examination showed a soft, poorly mobile, nonpulsatile mass involving the posterior neck and upper back, measuring approximately 33.0 × 20.0 cm. The lesion extended from approximately 2 cm below the external occipital protuberance to the inferior scapular angles. Multiple café-au-lait macules were present. Superficial sensation, cervical motion, and upper-limb motor function were preserved (Figure 1).

**Figure 1:**
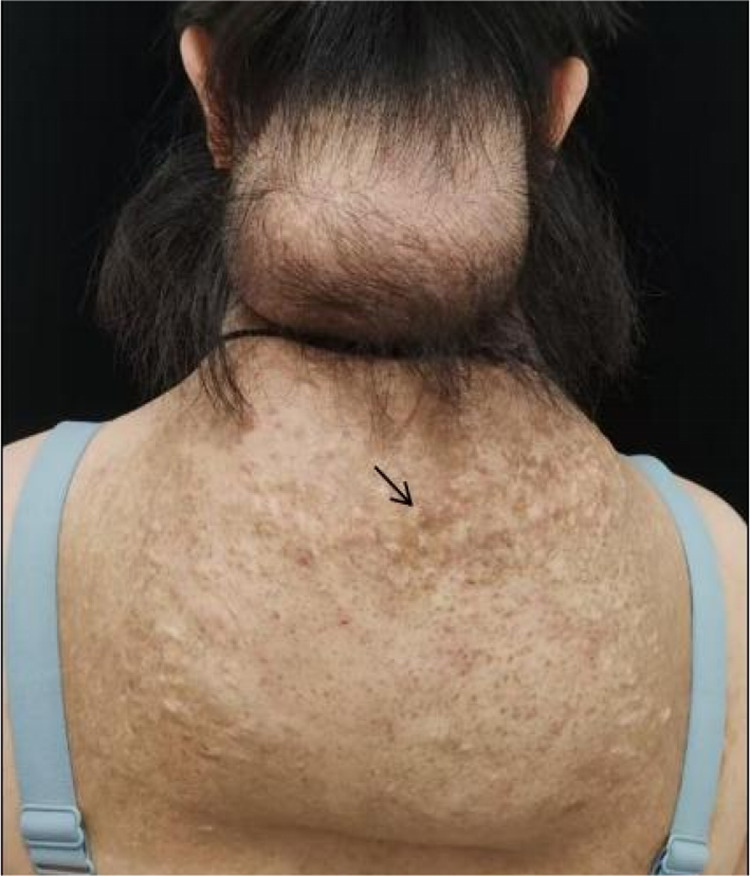
Preoperative appearance of the giant posterior neck and upper-back mass and café-au-lait macules (black arrows).

## Imaging and pathology

Magnetic resonance imaging showed diffuse thickening of the posterior cervicodorsal subcutaneous tissues and a heterogeneous lesion measuring approximately 28.1 × 5.1 × 16.8 cm, with tortuous internal vascular-like structures (Figure 2). The findings indicated a hypervascular soft-tissue lesion but were not specific for hemangioma.

**Figure 2:**
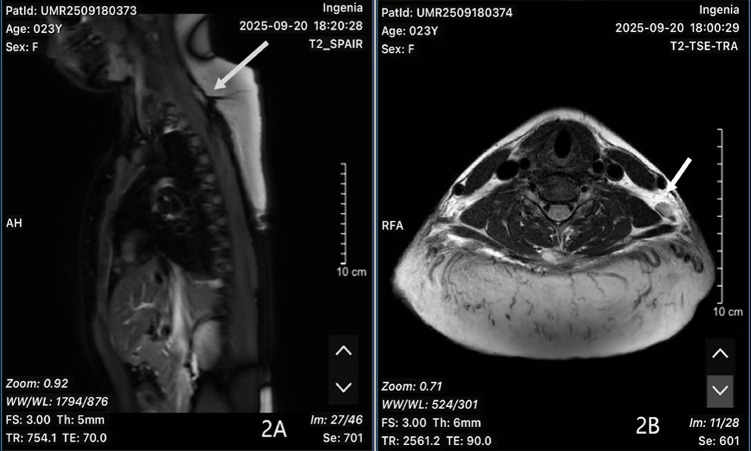
Preoperative MRI showing diffuse posterior cervicodorsal involvement and tortuous internal vascular-like structures. **(A)** Sagittal T2-weighted image showing the posterior cervicodorsal mass with internal vascular-like structures. **(B)** Axial T2-weighted image showing diffuse posterior cervical soft-tissue involvement.

The resected specimen consisted of gray-white to gray-yellow solid tissue measuring 26.0 × 24.0 × 7.5 cm, with focal hemorrhage. Histopathologic examination showed a benign neurofibroma. Immunohistochemistry demonstrated S-100 positivity, SOX10 positivity, scattered CD34 positivity, desmin negativity, and a low Ki-67 labeling index of approximately 1%, supporting the diagnosis and arguing against malignant peripheral nerve sheath tumor (Figure 3).

**Figure 3:**
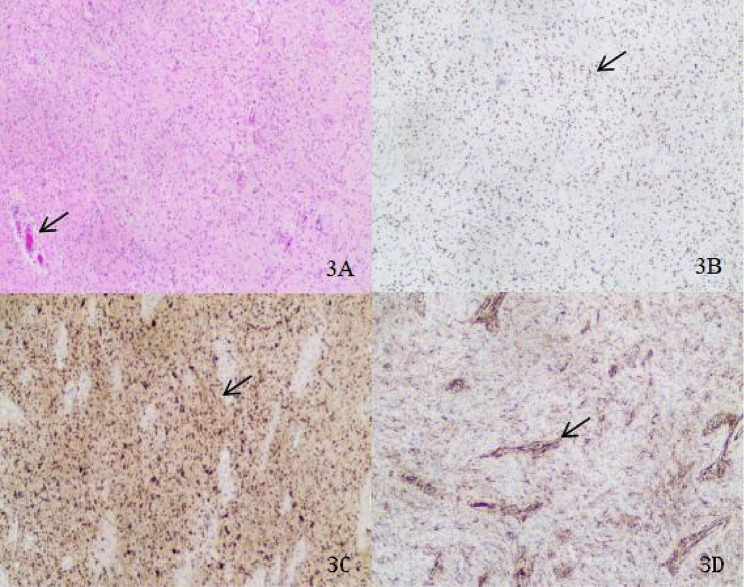
Spindle-cell neurofibroma with SOX10 and S-100 positivity and scattered CD34 positivity. **(A)** Hematoxylin and eosin staining showing loosely arranged spindle cells with myxoid stroma and intratumoral vascular structures. **(B)** SOX10 immunostaining showing nuclear positivity in tumor cells. **(C)** S-100 immunostaining showing diffuse positivity in tumor cells. **(D)** CD34 immunostaining showing positivity in intratumoral vascular structures and scattered tumor cells.

The differential diagnosis included a vascular malformation, diffuse or plexiform neurofibroma, fibromatosis, and malignant peripheral nerve sheath tumor. The clinical findings and immunophenotype supported an NF1-associated benign neurofibroma and did not show evidence of malignant transformation (1, 5, 18).

## Initial operation and failure of primary reconstruction

The recurrent tumor was excised under general anesthesia. Dissection proceeded circumferentially from the periphery toward the center along the superficial aspect of the deep fascia, avoiding direct entry into the lesion. Peripheral vessels were ligated sequentially and bipolar cautery was used for hemostasis. Macroscopic excision was achieved with an estimated blood loss of approximately 100 mL. The resulting defects measured approximately 15.0 × 16.0 cm in the neck and 35.0 × 22.0 cm in the back, corresponding to about 7% of total body surface area. Macroscopic excision was achieved. Owing to the large tumor size and intraoperative bleeding, 400 mL of blood was transfused during the procedure. The resulting defects measured approximately 15.0 × 16.0 cm in the neck and 35.0 × 22.0 cm in the back, corresponding to about 7% of the total body surface area.

For primary coverage, a split-thickness graft was harvested from areas of the tumor surface that appeared clinically preserved. This choice was intended to provide sufficient coverage while limiting creation of a second large donor wound. The graft was manually meshed at approximately 1:1.5, sutured to the defect, and covered with a single vacuum sealing drainage system at approximately −150 mmHg. Cefazolin was given perioperatively.

Fever developed on postoperative day 3, accompanied by bulging of the vacuum dressing and unstable suction. Exploration showed graft flotation over a pale-yellow subgraft fluid collection. Despite widening of the mesh openings and resealing, most of the graft became necrotic and detached; overall graft take was approximately 20%. Laboratory tests showed a white blood cell count of 9.67 × 10^9/L, neutrophils of 83.1%, an absolute neutrophil count of 8.03 × 10^9/L, and C-reactive protein of 57.51 mg/L. Wound culture grew Klebsiella pneumoniae, which was susceptible to cefuroxime and the other tested cephalosporins, carbapenems, amikacin, levofloxacin, tigecycline, and trimethoprim-sulfamethoxazole; intrinsic resistance to ampicillin was reported. The findings supported seroma-associated graft separation followed by secondary infection.

## Wound-bed optimization and salvage reconstruction

After transfer to our department in October 2025, necrotic graft remnants and devitalized tissue were removed. The wound underwent serial debridement, cleansing, silver-containing dressing care, and culture-directed antimicrobial treatment. A second operation was planned after exudate and malodor resolved, inflammatory findings improved, and repeat culture showed no pathogenic growth (Figure 4).

**Figure 4:**
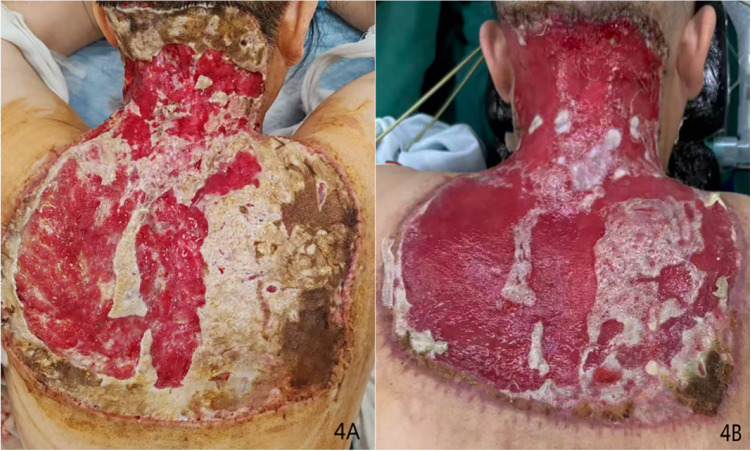
Wound after failure of the primary graft **(A)** and after wound-bed preparation before salvage grafting **(B).**

On October 22, 2025, excisional debridement was performed until a vascular granulation bed was exposed. A split-thickness graft was harvested from the upper lateral left thigh and mechanically meshed at 1:3 to cover the approximately 7% total body surface area defect. The graft was secured with sutures. Blood products were cross-matched and reserved preoperatively; however, no transfusion was required during the salvage procedure.

A silver-containing contact layer and foam dressing were applied over the graft. Because the wound crossed the curved and mobile neck-back junction, the negative-pressure dressing was divided into two independently sealed zones, one for the neck and one for the back. Multiple drainage tubes and reinforced sealing were used to improve fluid evacuation and maintain suction. Continuous pressure was adjusted within the clinically used range of approximately −125 to −200 mmHg.

Suction, drainage, and tube patency were checked daily. Low-pressure saline irrigation was used when needed to maintain tube patency. A cervical collar and thoracic-back brace limited neck and trunk movement and reduced shear. Cefuroxime, analgesia, fluid management, and nutritional support were provided. The negative-pressure dressings were removed on postoperative day 7, compression was continued according to graft condition, and sutures were removed on day 14.

## Outcomes and follow-up

The salvage graft was uniformly pink and adherent when the dressing was removed on postoperative day 7, without clinically evident hematoma, seroma, displacement, or necrosis. By day 14, the wound had epithelialized and the thigh donor site had healed (Figure 5).

**Figure 5:**
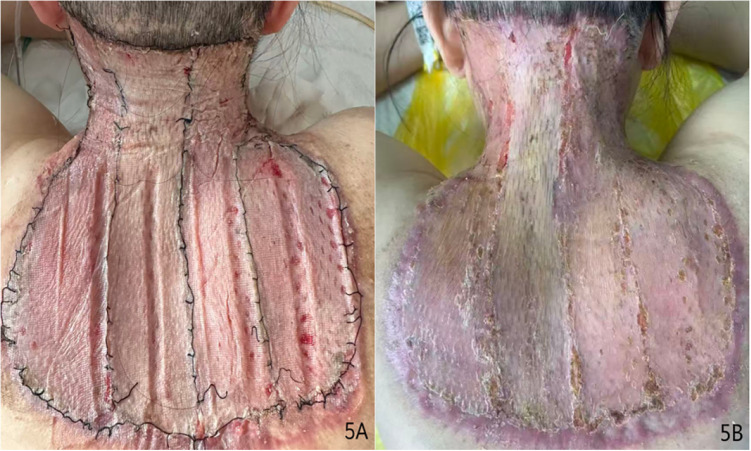
Salvage graft after drain removal **(A)** and at 2 weeks after suture removal **(B)**.

At 3 months, the graft was stable, sensation had largely recovered, neck motion was unrestricted, and the donor site was flat (Figure 6). At 6 months, no local recurrence was detected. The Vancouver Scar Scale score was 3 and the Neck Disability Index score was 5 (19, 20). The overall diagnostic and reconstructive pathway, the comparison between primary reconstruction failure and successful salvage, and the key technical elements of secondary wound reconstruction are summarized in Figures 7–9.

**Figure 6:**
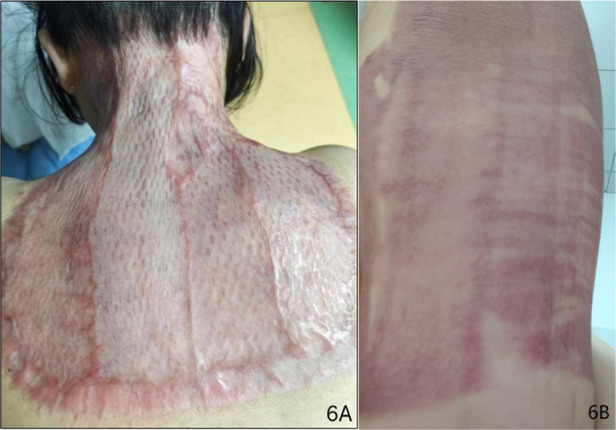
Three-month follow-up of the healed cervicodorsal graft **(A)** and left-thigh donor site **(B)**.

**Figure 7:**
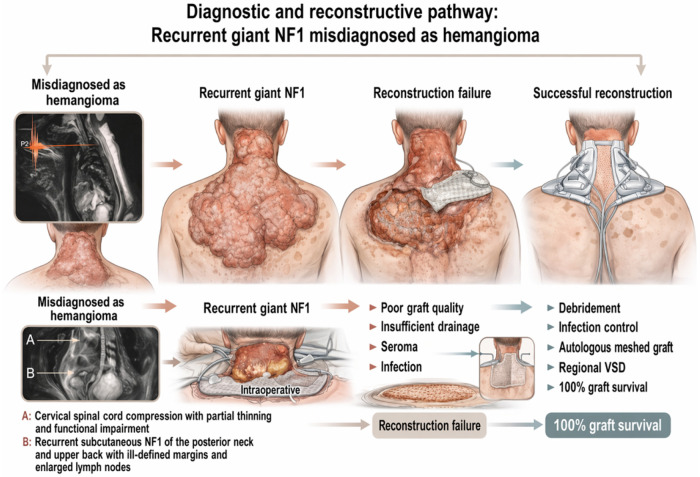
Schematic overview of the diagnostic and reconstructive pathway in a recurrent giant NF1 lesion initially misdiagnosed as hemangioma. The left panel shows the early phase, in which the posterior neck soft-tissue mass was misdiagnosed as hemangioma and MRI suggested internal abnormal vascular structures; the inset MRI panels labeled A and B show sagittal and axial views, respectively. The middle panel shows the recurrent giant NF1 lesion involving the posterior neck and upper back, scattered café-au-lait macules, and failure of the first reconstruction using tumor-surface meshed skin grafting with negative-pressure wound therapy because of poor graft quality, inadequate drainage, seroma formation, and secondary infection. The right panel shows successful reconstruction after complete debridement, infection control, autologous meshed split-thickness skin grafting, silver-containing dressing coverage, and regionally separated negative-pressure drainage for the neck and back.

**Figure 8:**
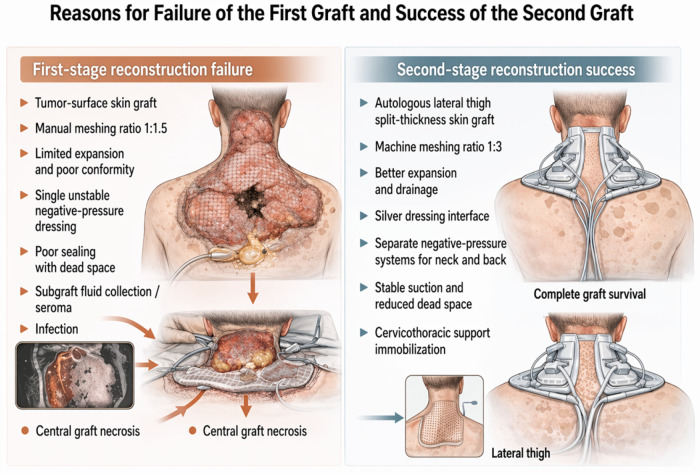
Schematic comparison of the causes of primary graft failure and the factors associated with successful salvage grafting. The left side illustrates the main contributors to failure after the first operation, including use of tumor-surface skin as graft source, manual 1:1.5 meshing, insufficient graft expansion and drainage, unstable single-circuit negative-pressure coverage, incomplete sealing with dead-space formation, subgraft fluid accumulation or seroma, secondary infection, and central graft necrosis. The right side illustrates the major factors associated with successful salvage, including healthy split-thickness graft harvested from the lateral left thigh, mechanical 1:3 meshing, improved graft expansion and drainage, silver-containing dressing coverage, regionally separated negative-pressure wound therapy for the neck and back, more stable suction with less dead-space formation, cervical and thoracic-back immobilization, and complete graft take.

**Figure 9:**
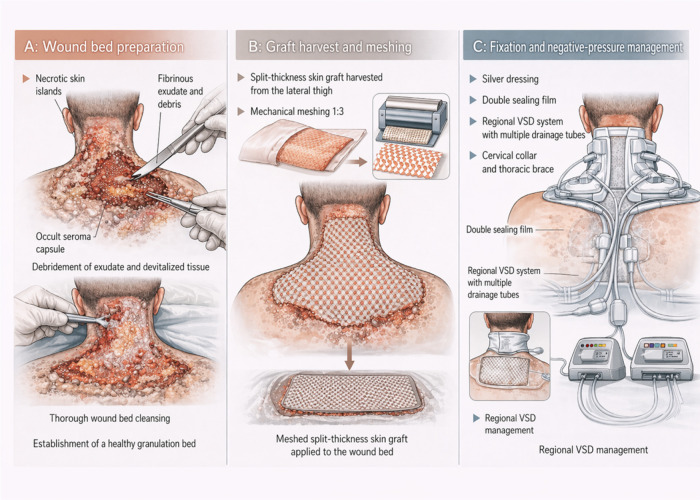
Key technical elements of secondary wound reconstruction. **(A)** Wound bed preparation, including complete removal of necrotic skin islands, fibrinous exudate, occult seroma cavity walls, and infected tissue to produce a well-vascularized granulation bed. **(B)** Autologous graft harvest and meshing, showing split-thickness graft harvest from the lateral thigh and mechanical 1:3 meshing before coverage of the cervicodorsal defect. **(C)** Fixation and negative-pressure management, showing silver-containing dressing placement over the graft, independent negative-pressure coverage of the neck and back with multiple drains and double-layer sealing, and adjuvant cervical and thoracic-back immobilization to improve graft survival and wound healing.

## Discussion

This case illustrates how an initial diagnostic error and an unfavorable recipient bed can complicate treatment of a giant recurrent neurofibroma. Hypervascular imaging and intraoperative bleeding may suggest a vascular lesion, but these features are not diagnostic. Current NF1 assessment integrates clinical criteria, imaging, pathology, and tumor surveillance (1, 4–6, 8, 15, 18). In this patient, dissection along a preserved deep-fascial interface and stepwise vessel control were associated with limited blood loss; however, this observation from one superficial cervicodorsal lesion should not be interpreted as evidence that the same approach will be blood-sparing in all giant neurofibromas (11, 12, 17).

The initial use of skin from the tumor surface requires particular caution. Its practical rationale is clear: it can provide a large amount of tissue and reduce donor-site morbidity, and recent case literature describes this option in selected giant neurofibromas (16). Nevertheless, diffuse or plexiform neurofibromas may extend into the dermis and subcutaneous tissue without a sharply defined capsule. Clinically normal-appearing overlying skin may therefore contain microscopic tumor or altered neurovascular tissue. Reimplantation could theoretically transfer residual lesional tissue, compromise oncologic margin assessment, or complicate later surveillance. Abnormal dermal architecture or vascularity may also make the graft less reliable biologically. Histologic assessment of candidate skin and confidence in tumor clearance should therefore precede reuse; healthy distant donor skin is preferable when oncologic or biological suitability is uncertain (16, 18). In the present case, the immediate causes of failure were subgraft fluid, unstable fixation, and infection, and the available data do not prove that the graft source itself caused necrosis.

The salvage procedure used established principles rather than a new reconstructive method: removal of necrotic tissue, control of infection, preparation of a vascular wound bed, regrafting with healthy autologous skin, drainage of fluid, and limitation of shear. Wider 1:3 meshing increased coverage and drainage but may trade improved expansion for a more conspicuous mesh pattern (21, 22). Negative-pressure wound therapy is supported as a graft bolster, including in irregular and high-mobility areas, where it can reduce displacement and fluid accumulation (23–28). The two-zone neck and back arrangement in this case should be viewed as a practical dressing modification used to improve sealing and drainage across a large curved surface, not as a novel reconstructive principle. The cervical collar, back brace, and subsequent compression were also important because graft adherence depends on both fluid control and immobilization. Silver dressing use was adjunctive and should not substitute for debridement and culture-directed treatment (29, 30).

The interpretation is limited by the single-patient design, 6-month follow-up, lack of germline NF1 testing, and absence of direct histologic evaluation of the harvested tumor-surface skin. Several variables changed simultaneously between operations, including the wound bed, infection status, graft source, mesh ratio, dressing configuration, and immobilization. The successful outcome therefore cannot be attributed to the divided negative-pressure dressing alone, and no comparative recommendation can be made.

## Conclusion

A recurrent giant neurofibroma may resemble a vascular tumor and can leave a difficult defect after excision. In this patient, standard salvage measures—repeat debridement, infection control, healthy autologous meshed grafting, secure negative-pressure fixation, compression, and immobilization—produced wound closure after primary graft failure. Dividing the dressing into neck and back zones was a case-specific practical adjustment; its independent benefit and generalizability require further study.

## Data Availability

The raw data supporting the conclusions of this article will be made available by the authors, without undue reservation.
